# An Education Framework for Effective Implementation of a Health Information System: Scoping Review

**DOI:** 10.2196/24691

**Published:** 2021-02-24

**Authors:** Tharshini Jeyakumar, Sarah McClure, Mandy Lowe, Brian Hodges, Katharine Fur, Mariquita Javier-Brozo, Maria Tassone, Melanie Anderson, Tim Tripp, David Wiljer

**Affiliations:** 1 University Health Network Toronto, ON Canada; 2 Institute of Health Policy, Management and Evaluation University of Toronto Toronto, ON Canada; 3 Department of Occupational Science and Occupational Therapy Faculty of Medicine University of Toronto Toronto, ON Canada; 4 Department of Psychiatry Faculty of Medicine University of Toronto Toronto, ON Canada; 5 Wilson Centre Toronto, ON Canada; 6 Department of Physical Therapy Faculty of Medicine University of Toronto Toronto, ON Canada; 7 Office of Education, Centre for Addiction and Mental Health Toronto, ON Canada

**Keywords:** health information system, health care providers, education, learning, patient care

## Abstract

**Background:**

To optimize their use of a new Health Information System (HIS), supporting health care providers require effective HIS education. Failure to provide this education can significantly hinder an organization’s HIS implementation and sustainability efforts.

**Objective:**

The aim of this review is to understand the most effective educational strategies and approaches to enable health care providers to optimally use an HIS.

**Methods:**

Ovid MEDLINE, Ovid Embase, EBSCO Cumulative Index to Nursing and Allied Health Literature, and EBSCO Education Resources Information Center were searched to identify relevant papers. Relevant studies were systematically reviewed and analyzed using a qualitative thematic analysis approach.

**Results:**

Of the 3539 studies screened, 17 were included for data extraction. The literature on the most effective approaches to enable health care providers to optimally use an HIS emphasized the importance of investing in engaging and understanding learners in the clinical context, maximizing the transfer of learning to care, and designing continuous and agile evaluation to meet the emerging demands of the clinical environment.

**Conclusions:**

This review supports the advancement of a new HIS learning framework that organizational leaders and educators can use to guide HIS education design and development. Future research should examine how this framework can be translated into practice.

## Introduction

### Background

Health Information Systems (HISs) have been proposed as one solution in a multipronged organizational approach to transforming the quality of care delivered, increasing patient safety, and reducing health care costs [1]. An HIS is defined as a system designed to integrate data collection, processing, and reporting and the use of health information to influence policy making and improve health service effectiveness and efficiency [2]. This enables the facilitation of health information sharing among multiple authorized custodians across the health care continuum in support of clinical efficiency and optimal quality care [3]. Challenges to HIS implementation can result from staff and care providers being unfamiliar with the system features and functionalities, thus encumbering the ability of staff and providers to use HIS in their work environment most effectively. Given the rapid pace of adoption of digital HISs globally and the skills needed to effectively use HISs in practice, it is crucial to support and educate health care providers at all levels, across all areas of the health system, and as close to implementation as possible, on how the HIS system can be leveraged to improve clinical practice. Simply teaching the system is not sufficient to successfully enable learners (entry-to-practice and practicing health care staff) to use new technology.

As health care organizations respond to budgetary, regulatory, and societal pressures to implement an HIS, care providers are confronted with an ever-increasing technology-enabled care environment [4]. Paradoxically, although the implementation of HISs is meant to create efficiencies, their widespread diffusion and accompanying complexity have been associated with a growing recognition of clinician dissatisfaction and burnout [5]. However, health care providers see education as integral to use technology successfully [4]. On the basis of the model of skill acquisition, Bredfeldt et al [1] determined that educating novices entails more than just imparting knowledge. Further, it is essential to provide education that is pertinent to address learning needs and provide an opportunity to learn the nature of the real setting and its associated variability [1]. It is also imperative to acknowledge learners’ expertise and engage them throughout all phases in the development of the educational program [6]. This may be especially true for health sciences students and trainees who may have deeper knowledge of using various technologies, thereby having the potential to play a role of educator or facilitator with supervisors in practice. Furthermore, education may enable care providers and staff to understand the concepts underlying the different HIS tasks as the emerging needs of their clinical practice continuously evolve rather than simply learning the features of an HIS. HIS education can also influence user adoption and the ability of health care providers and staff to effectively use the technology [4]. On the basis of learners’ feedback in the study, McAlearney et al [7] noted that staff who received excellent education and hands-on experience with an information technology system adopted realistic expectations and achieved a sense of control within the HIS environment. Adequate education and support, technology literacy, and overall competencies of health providers were identified as critical factors in HIS implementation.

Many information technology users encounter a steep learning curve at the initial stages of implementation and can take several years to become an expert in the features and functions of a system [8]. McLean et al [8] suggested that users’ attitudes toward system use in the initial stages could be leveraged to gain valuable insights into how the system will be used in the later stages. Thus, education remains a critical component in understanding the benefits of HISs and attaining value-added use, particularly during the adoption or early stages of implementation [8,9]. Furthermore, to enable ongoing learning in using an HIS adeptly, education strategies will need to be evaluated and refined over time, as education will need to continuously evolve to meet users’ needs and comfort level with the HIS [8,9].

In several HIS implementation projects, inadequate HIS education has led to challenges in the adoption and suboptimal use of the system. An example is an electronic health record (EHR) implementation project at Cedars Sinai Hospital, California, where a dearth of staff education contributed to poor adjustment to the new system and created a sense of fear and apprehension [10,11]. Consequently, this lack of education threatened staff autonomy and eventually contributed to the rejection of the HIS and project failure [10,11].

### Objectives

As HISs become an integral part of patient care, building an effective educational strategy may ultimately aid in the successful implementation and sustainment of an HIS. Recognizing the importance of education programs in supporting HIS implementations, this study was conducted to understand the current state of HIS education programs as reported in the academic literature. Specifically, the objective of this study is to establish a foundational understanding of the most effective strategies and approaches to enable individuals to optimally adopt and effectively use and learn from an HIS, both during and postimplementation.

## Methods

### Overview

**Figure 1:**
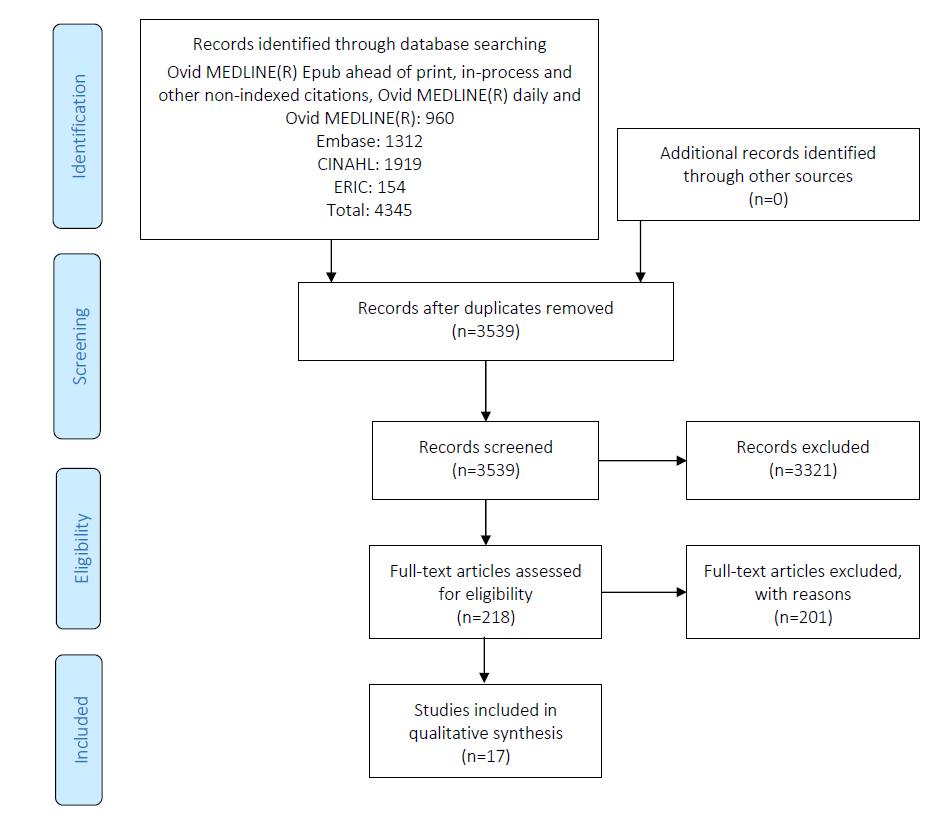
PRISMA (Preferred Reporting Items for Systematic Review and Meta-analysis) flow diagram of scoping review results.

A scoping review methodological framework adopting the approach by Arksey and O’Malley [12] was used to enhance the reproducibility and reliability of our findings. One of the goals of this approach was to broadly examine a topic area to map key concepts, evidence types, and current gaps in research in a well-defined field using a wide array of literature. This was an ideal starting point to better understand the landscape of research within a specific subject area. To illustrate the scoping review process, the PRISMA (Preferred Reporting Items for Systematic Review and Meta-Analysis) diagram [13], shown in Figure 1, and the PRISMA scoping review checklist, which outlines the important milestones of a scoping review, were used [14] (Multimedia Appendix 1).

### Stage 1: Identifying the Research Questions

To establish a baseline understanding of the most effective strategies and approaches to enable individuals to optimally use an HIS system in academic literature, this study sought to answer the following questions:

1. What education approaches led to an effective HIS implementation? What are the reported results of classroom-based, web-based, and blended learning in terms of educating staff on HISs?
2. What are the measures and outcomes used to assess the effectiveness of education and its impact on the implementation of the HIS?
3. What are the most effective approaches for enabling individuals to optimally use an HIS? How is education most effectively delivered?

### Stage 2: Identifying Relevant Studies

An iterative process was used to design effective database strategies to identify eligible papers, involving several discussions with information specialists and the research team at our institution. Strategies, including subject headings, keywords, and related terms for HISs; education approaches; and training modalities were designed by a health sciences librarian for each of Ovid MEDLINE, Ovid Embase, EBSCO Cumulative Index to Nursing and Allied Health Literature, and EBSCO Education Resources Information Center and results were downloaded in November 2019 (Multimedia Appendix 2). No date or language limits were applied. HIS was defined in these searches as an electronic medical record (EMR), EHR, medical records systems, computerized medical records, electronic patient records, computer health records, computer medical records, computer hospital records, clinic information systems, electronic health medical records, and electronic hospital medical records. Relevant studies were identified through a title and abstract scan and confirmed via a full-text review. For the study selection, see the PRISMA diagram (Figure 1).

### Stage 3: Study Selection

Peer-reviewed journal papers eligible for inclusion met the following criteria: (1) examined educational approaches (ie, classroom, instructor-led, web-based training, e-learning, and hybrid learning), (2) discussed HIS systems (EMR, EHR, Clinical Information system, etc), (3) discussed the effectiveness of different approaches in educating staff to use the HIS, (4) described an educational program related to HIS, and (5) ensured education be conducted in a hospital setting. Initially, only English papers were included; however, as there were no papers found in other languages, no papers were excluded. All study designs, whether quantitative or qualitative studies and papers found in reports and research papers except for viewpoint papers, such as editorials, were included. Studies conducted in an academic setting and papers that did not meet the aforementioned criteria or describe an HIS system were excluded. Five coauthors independently reviewed the paper to determine each study’s eligibility, and in cases of uncertainty, a senior reviewer with expertise in the topic of HISs was consulted.

### Stage 4: Data Items and Data Collection Process

A standardized charting form was developed to capture the following domains: study details (type of study, year, and country), the objective of the study, study design (if applicable), study participants, intervention, study outcomes, and main results of the study. The PICO (Patient Problem or Population, Intervention, Comparison or Control, and Outcome) framework was used to capture details of the study, where the study outcomes were categorized using the Kirkpatrick-Barr [15] framework of educational outcomes, shown in Textbox 1. This framework was selected, as it provided a standardized method of categorizing the type of educational outcomes reported by each paper.

**Kirkpatrick-Barr framework of educational outcomes.**

**Level 1: Learners’ reaction**

- Learners’ perspectives on the learning experience and satisfaction with the educational program [15]

**Level 2a: Modification of attitudes and perceptions**

- Changes in attitudes and perceptions toward patients or clients and their condition, circumstances, care, and treatment [15]

**Level 2b: Acquisition of knowledge and skills**

- Changes in knowledge and skills [15]

**Level 3: Change in behavior**

- Changes in behavior of participants’ transfer of learning to their practice setting and changed professional practice [15]

**Level 4a: Change in organizational practice **

- Wider changes in the organizational practice and provision of care as a result of an education program [15]

**Level 4b: Benefits to patients or clients**

- Improvements in health or well-being of patients or clients attributable to an education program [15]

### Stage 5: Synthesizing and Reporting the Results

A qualitative *narrative review* approach was adopted, and the authors independently reviewed all 17 studies. The educational outcomes reported in each paper were categorized using the Kirkpatrick-Barr framework, which also helped inform the thematic analysis. The findings were synthesized, and a thematic analysis approach was used to critically analyze the papers and develop a coding structure. Emerging themes were identified, compared, and consolidated by 3 authors. The consultation phase provided an opportunity to validate the findings and critically examine the inconsistencies or lack of clarity evident across the papers reviewed. Discussions and consultation with content experts within our team enabled us to further iterate and contextualize the themes.

## Results

### Search Results

The initial database search yielded 4345 papers. Once duplicates were removed, titles and abstracts of 3539 unique citations were identified. We screened these papers and identified 218 citations based on broad relevance to the topic area. The 218 abstracts then went through the second round of scrutiny against the inclusion criteria, and 33 papers were selected for full-text review. Following further inspection, 16 papers were excluded, as they did not meet the inclusion criteria. Table 1 describes the characteristics of the studies included in this study.

**Table 1:** Study characteristics (N=17).

Study characteristics		Value, n (%)	References
**Country of publication**			
	United States	12 (70)	[1,4-7,16-22]
	Australia	1 (6)	[23]
	Namibia and Tanzania	1 (6)	[24]
	United Kingdom	1 (6)	[25]
	Denmark	1 (6)	[26]
	The Netherlands	1 (6)	[27]
**Research method**			
	Literature review	3 (18)	[25-27]
	Questionnaire or survey	4 (23)	[6,19,21,23]
	Semistructured interview	1 (6)	[9]
	Mixed method	6 (35)	[4,5,16,17,20,22]
	Quasiexperimental	2 (12)	[1,24]
	Case report	1 (6)	[18]
**Year of publication**			
	2016-2019	5 (29)	[5,23-26]
	2010-2015	9 (53)	[1,4,6,7,16,17,19,22,27]
	2006-2009	3 (18)	[18,20,21]
**Education approach^a^**			
	In-classroom training	5 (38)	[5,6,16,18,20]
	e-Learning	1 (8)	[22]^b^
	Blended learning	5 (38)	[1,17,19,23,24]
	Classroom versus blended learning	1 (8)	[4]
	Simulation training	1 (8)	[19]

^a^Literature reviews were excluded.

^b^Did not provide evaluation outcomes.

### Research Question 1: Reported Results of Classroom-Based, Web-Based, and Blended Learning in Terms of Educating Staff on HISs

On the basis of the thematic analysis, this study identified 3 major themes across the different educational approaches that led to a more effective HIS implementation:

- Invest in engaging and understanding learners in the clinical context
- Maximize the transfer of learning to care
- Continuous and agile evaluation designed to meet the emerging demands of the clinical environment

In addition, the themes addressed in this study encompass fundamental elements that may be used to guide educational design and support developmental expertise in clinical environments (Table 2).

**Table 2:** Elements associated with the 3 major themes identified in the thematic analysis.

Theme and element		Definition of element	Studies using an element
**Invest in engaging and understanding learners in the clinical context**			
	Assessment of individual, team, and organization capabilities	Assess appropriate learning needs Assess computer literacy Ascertain clinical background and role Provide education based on customized workflows (training based on the work environment and department needs, eg, inpatient provider, ambulatory provider)	[1,16,21,23,26,27]
**Maximize the transfer of learning**			
	Practice and problem-based learning	Use a problem-based approach to learning instead of task-based learning (ie, learning built on predefined tasks addressed in clinical practice). Integrate hands-on experience to enable learner empowerment.	[1,4,6,7,17,23,25,28]
	Integrate learning into practice	Create opportunities to integrate HIS learning into practice. Engage learners in their clinical context. Employ simulation- and scenario-based learning techniques (real-world uncontrived experience). Provide opportunities to create tools and items that can be used in clinical practice (eg, creating personal preference lists of frequently used orders, creating a patient-specific care plan).	[1,4,16,22,25,26]
	Enhance practice improvement and performance	Ensure the learning time is as close to the launch of the HIS as possible. Adopt longitudinal approaches to training. Identify and integrate super users as part of education planning. Collaborate with and learn from clinical champions.	[6,7,17,23,25,26]
**Continuous and agile evaluation designed to meet the emerging demands of the clinical environment**			
	Evaluation and feedback	Being agile to meet the emerging demands of the clinical environment Understanding how health care providers perceive HIS education and eventually its application Adaptability and enhancement of curriculum revisions	[5,6,21]

#### Invest in Engaging and Understanding Learners in the Clinical Context Theme

The *Invest in engaging and understanding learners in the clinical context* theme includes both understanding learners’ needs and understanding what incentives may best enable learner participation in HIS education.

Several papers have identified the importance of assessing the current capability level of learners in terms of clinical and technical skills to tailor learning accordingly, including providing adequate staff and time for education. Many health care providers received their education as health care providers before information technology became ubiquitous and, therefore, may lack the essential technical skills required to use an HIS effectively [1]. Benwell et al [23] reported that learners often felt that the HIS training sessions failed to address their learning needs, as the program was either too simplistic or advanced. This point was further reinforced in a study that examined traditional forms of education approaches, assuming that each health care provider shared the same knowledge and skill level. However, the authors discovered that the skillset of care providers differed based on their experience and educational levels [26,27]. In another peer-reviewed paper, the educational team at a hospital designed a course for health care providers with average computer skills.

The curriculum timing was based on an end-user with average computer skills: someone able to use a mouse and familiar with windows functionality. The design was frustrating to the advanced computer users because they were ‘‘slowed down’’ and frustrating to students with minimal skills because content was covered much too fast. Most staff attended training in 8-hour blocks, compounding this issue. Evaluations indicated that the number of hours spent in the classroom was over-powering and not conducive to learning. Staff reported feelings of burned out and too much content to absorb.

21

Previous experiences with computers and the perceptions that learners bring with them regarding the value of technology influence their receptiveness during training sessions [21]. Edwards et al [4] stated that pretraining enabled those health care providers who needed it to gain baseline familiarity and improved their performance. Furthermore, Bredfeldt et al [1] reported:

Trainees who are proficient at problem list management may have already reached the functional ceiling, leaving no room for improvement. In contrast, while some training participants were very practical at medication list management, there was still room for improvement.

1

Adequate technology literacy and general competencies of health care staff have been identified as critical factors for implementing HIS systems [16,26]. Without these, further education sessions may be required to support health care providers before they engage in learning specific to HIS.

Four papers discussed the significance of providing incentives to help health care staff expand their information technology competencies to encompass new skills [1,6,17,18]. A study by O’Brien [18] asserted that to cover the expense of adequate training and replacement nurses, the organization provided incentives to staff to complete their training on days they were not scheduled to work. In addition, the organization has developed an incentive program to encourage staff to fill in for colleagues who were participating in training or to attend a training class in the evening or weekend at full pay. In another study, providers were recognized for their time by being eligible for continuing medical education credits [1,6].

#### Maximize the Transfer of Learning to Care Theme

The theme of *Maximize the transfer of learning to care* encompasses providing hands-on practice, integrating real-life case scenarios, engaging key stakeholders and staff champions, and scheduling education sessions close to the actual use of the system, all of which contribute to a learner-centric approach and a more successful HIS implementation (Table 2).

Many papers stressed the importance of providing learners with significant amounts of time to engage in hands-on activities [4]. Interaction with the HIS was a key priority among all participants across several studies [4,6,17,25]. Participants were provided with an overview of the key features of the system, using a combination of lectures and practical exercises, thus enabling the learners to gain hands-on experience using the new system [25]. In addition, hands-on activities enabled health care providers to gain more practice and become familiar with the system and have an opportunity to ask questions [6].

Class participants indicated that the hands-on exercises were the most useful portion of the class, and they appreciated the ability to build things in class that could be used in the clinic.

1

This finding corresponds with concerns from health care managers that learners should be competent in using HIS in their work setting [4]. By providing an opportunity to deliberately practice with the system, active learning is encouraged, thereby increasing learners’ confidence [4]. Nicklaus et al [17] described that hands-on experience allows for the practical application of the concept and enables distraction-free instruction. This approach encourages learners to set the pace of their learning without becoming overwhelmed with a lot of content in a short period of time while increasing their confidence and competency levels [17]. By promoting a self-regulated learning principle, effective learning can be achieved by empowering learners to take part in their own learning process, set their own goals, and challenge their critical thinking skills [17]. Self-regulated learning is defined as a cyclical process that enables the learner to guide their goal-directed activities, evaluate their performance, and then reflect on the outcomes [28]. McAlearney et al [7] asserted the following:

...training programs that include opportunities for learners to observe others using the EHR system, and those that provide active learning opportunities, should enhance the learning process because they give learners opportunities to develop positive perceptions about their own abilities related to using the EHR.

7

This point was reinforced in a study by Bredfeldt et al [1], stating that classroom-based training and hands-on activities were associated with the improved utility of using the new system. The authors noted that a live EHR environment allowed staff and clinicians to build tools they could use when they returned to the clinic and the use of ancillary resources [1]. Through continuous interaction with the system, learners reduced their cognitive effort associated with performing the task [16].

In addition, several studies have found that educational programs that incorporate real case scenarios, with an emphasis on clinical workflow enhance outcomes [25,26]. Interactive scenarios presented with a mini case study highlighted the importance of new HIS elements, enabling the care providers to better understand the new updates involved with the HIS and the manner in which they needed to document [22]:

A hybrid teaching method that entailed both e-learning and a supplemental education session providing face-to-face personal communication, case examples, and examples of errors improved timeliness, completion and accuracy of nursing documentation significantly.

16

Studies have highlighted the importance of developing expertise-specific scenarios that are relevant to health care providers [25]. The use of various approaches may appeal to individual learning needs, with learners appreciative of relevant clinical scenarios in particular [25]. The scenarios were designed to reflect the daily workflow process and enabled learners to visualize how the HIS can potentially be used in their work environment [22]. Furthermore, the scenarios provided each learner with further exposure to the workflow and an opportunity to critically reflect through the different processes [22].

The literature findings described the importance of and positive changes in engaging key stakeholders and staff champions in achieving targeted organizational change. Super users (expert users who have received supplementary education and are capable of educating other staff) were found to play a critical role in providing unit-level assistance and reducing the need for expensive external education and training [18,25]. They act as facilitators in each area or department, supporting and educating new staff [18,26]. McAlearney et al [7] reported:

...when learners observe others successfully using the EHR, their efficacy expectations are increased because of their corresponding beliefs that they also possess the capabilities to master the EHR system.

7

The authors emphasized the importance of positive behavior modeling (role modeling from peers in the work environment), which demonstrates effective approaches to help overcome these challenges. They acknowledged that engaging champions and super users may foster transformative learning and contribute to a learner-centric approach. In a study on training programs that leverage the skills of super users, it was reported that they contribute to better learning outcomes and meaningful use of the HIS [7]. Pantaleoni et al [6] described that the success of the training program was also attributed to the lead physician, as they provided guidance on the clinical context and knowledge of institutional workflows, such as the number of distinct provider workflows and how to group providers in a training session [6]. In addition, physicians were involved in the design and delivery of the training communication for hospital and staff leadership [6]. Pantaleoni et al [6] asserted that it is vital that the super user has an interest in education, institutional knowledge, and good communication skills.

In addition to the engagement of organizational stakeholders and staff champions, formalized education scheduled close to the actual use of the system was identified as beneficial to end users [23,26]. The study findings suggested that health care providers benefit from formal education only when it occurs in close proximity to their use of the HIS. Pantaleoni et al [6] emphasized the following:

Training classes should be offered 2 to 8 weeks prior to the change. Training that occurs greater than 8 weeks will likely not be remembered by the end-user.

6

It has been noted that education delivered too early or too late could potentially waste resources and raise frustrations among staff [23,26]. Moreover, the authors stated that following 30 days of unit-based experience, most staff ultimately exhibit a similar skill level. Education that is scheduled in close proximity to the time of end-user use may facilitate the greatest impact on performance and knowledge acquisition [23,26]. Furthermore, formalized (instructor-led) education may not be needed for all learners, as some participants reported formal education to be inefficient and of little value; however, daily exposure to the HIS improved their performance [23].

#### Continuous and Agile Evaluation Designed to Meet the Emerging Demands of the Clinical Environment Theme

A review of the studies suggested that continuous evaluation supports an agile approach to meet the emerging demands of the clinical environment (Table 2).

Pantaleoni et al [6] described the need to conduct an evaluation of an HIS educational program:

We then conducted an evaluation of a pilot implementation of the eLearning course to ensure that the resources matched needs; were understandable, usable, and useful; and contributed to quality improvement of future HIS eLearning resources.

6

Furthermore, McCain [21] stressed the value of evaluating educational programs continuously to identify course strengths and weaknesses in stimulating curriculum revisions. The authors emphasized that all HIS-related training and education programs should be continually updated to stay abreast of the evidence base and innovations.

Future work must include expansion and optimization of the current modules, and targeted dissemination to support uptake in appropriate settings. If evidence-based strategies for training providers Health Information Technology (HIT) are lacking, appropriate and effective use of these technologies will be limited, and many costly and potentially powerful HIT projects may fail to improve the quality of healthcare.

21

Hence, the evaluation allowed educators to understand the need for change in work processes and practices and an opportunity to establish mechanisms to share learning across the organization.

Importantly, although continuous evaluation can lead to learner-centric education delivery, HIS data can also be leveraged to prioritize interventions for system optimization and workflow redesign and to identify struggling learners who may require additional training or support [5]. In a study by Kadish et al [5], clinicians requested individualized training after several rounds of group training to improve their own efficiency in the EMR. Ensuring that the educational content was relevant and applicable to all learners was challenging, as individual skill sets in using the system varied among care providers. This study accentuated the importance of capturing data over time to inform continuous and personalized assistance to optimize the use of the HIS after initial training. Individualized education ensured that educators were able to adapt the content to accommodate the diversity of clinical practice at the individual and group levels to improve the competency and confidence of learners in the use of HIS to find clinical information [5].

### Research Question 2: Measures and Outcomes Used to Assess the Effectiveness of Education and Its Impact on the Implementation of HIS

Twelve papers presented the results of their training evaluation [1,4-6,16-21,23,24]. As training approaches and outcomes varied across studies, each approach will be briefly discussed (Table 3), followed by measures and statements of education outcomes associated with each educational approach. The classification of educational outcomes will be guided by Kirkpatrick-Barr.

**Table 3:** Summary of the 12 studies that assessed the effectiveness of the education program.

Training approach and author			Measures		Outcomes
**Classroom training**					
	Pantaleoni et al [6]	Survey assessed providers’ overall training experience, including trainer preparedness, course design, handouts, and the learner’s overall readiness to use the system		High physician satisfaction with the program *(level 1)* Positive effect on confidence in knowledge acquired *(level 2a)*	
	Kadish et al [5]	Providers were sent 2 surveys: The first survey was sent before training and used a 5-point Likert scale to measure confidence in the EMR^a^ overall and in 5 key activities. Immediately after training, a second survey was sent to participants to evaluate the session and to gauge confidence in the same activities. Changes in time spent in various EMR activities before and after training were compared using a paired Wilcoxon test.		Participants reported an increase in confidence across all activities *(level 2a)*, and almost all providers agreed that the training enhanced their efficiency (perceived; *level 3).* A reduction in the overall time in the EMR system was observed. Participants reported becoming more efficient with the use of the EMR *(level 3).*	
	Evatt et al [16]	Nurses completed a knowledge and attitude survey before and after education session: 10-item researcher-designed instrument Questions assessed knowledge regarding timeliness policy, area content, and information within areas Likert scales: testing attitude toward the completion of the EHR^b^ nursing admission assessment Sampled charts of patients admitted to the 2 units before and after completion of the sessions: To evaluate the timeliness of completion, the time (in minutes) from patient admission to the unit to submission of the nursing admission assessment to the EHR was determined. Accuracy of documentation was assessed with regard to the accuracy of the past medical history.		Nurses’ attitudes *(level 2a)* and knowledge *(level 2b)* regarding completion of the EHR nursing assessment admission assessment improved significantly. Following the educational session, the mean time to completion of the EHR nursing admission assessment decreased *(level 3).* Timeliness, completeness, and accuracy of assessment documentation (after the session) were improved significantly after use of a hybrid approach *(level 3).*	
	O’Brien [18]	Physician survey was conducted 2 months after the physician order-entry go-live date.		90% agreed that the EMR system made it easier for them to do their work *(level 1).* Medication errors caused by illegibility and transcription were eliminated completely *(level 3).* Staff also have found that the EMR system makes their jobs more efficient *(level 3).* Patient satisfaction scores for the overall satisfaction with care climbed to their highest levels *(level 4b).*	
	Kraus et al [20]	Measure the level of adoption of CPOM^c^: percentage of the utilization of HIS^d^ by CPOM of physicians.		Physician adoption reached the first-year goal of 40% physician entry in the first month and stabilized at 75% within a year. The impact has been noted in pharmacy, where the average time from order to pharmacist verification decreased from 90 min before CPOM to 17.9 min a year later *(level 3).* The resulting order sets and their increasing use in clinical care can be considered another measure of success.	
**Blended learning**					
	Bredfeldt et al [1]	Evaluated 2 outcome measures in the EHR data for 6 months before and after training: Proportion of visits in which either the problem list or the medication list was modified Modifying the problem list included adding or deleting problems from the problem list or attaching comments to existing problems on the list Modifying the medication list included marking medications as chronic, removing inactive medications, or marking the medication list as reviewed		Training was related to a small but significant increase in the use of key EHR capabilities: Participants increased their use of both the problem list from 22% to 24% of visits and their use of the medication list from 41% to 45% of visits after the education session *(level 3).*	
	Benwell et al [23]	Questionnaire (10-point Likert scale and dichotomous scale) To rate their self-perceived skill level using computers in general versus BOSSnet (digital medical record), the usefulness of the ICT^e^ training session and their willingness to train others The time taken to complete all tasks (efficiency) and the number of incorrect mouse clicks (accuracy) used to complete each task were recorded during the education session.		A significant improvement in both efficiency and accuracy for all participants during the session was observed *(level 2b).* The greatest improvements in task performance followed daily ward–based experience using BOSSnet rather than formalized training.	
	Nicklaus et al [17]	Observations to measure the effectiveness of training		Learners were satisfied with the learning laboratory as it provided an opportunity for them to practice and understand the system *(level 1).* They reported that scenario-based practice time stimulated realistic documentation and that they began to better comprehend the information.	
	Rudd et al [24]	The primary evaluation outcome was knowledge gain resulting from the completion of the blended e-learning course, measured by differences in posttest and pretest scores. Secondary outcomes included achievement of a 70% passing score and participant satisfaction with e-learning module content, format, and delivery.		Respondents reported high satisfaction with the overall content of the course and with the e-learning modules *(level 1).* Blended e-learning course participants gave positive feedback about the course structure *(level 1),* and their knowledge of HIS competencies. Participants experienced strong learning gains in both, although learning gains were somewhat greater in the in-person course (level 2b).	
	McCain [21]	Likert scale and open-ended questions Rating how well objectives were met and clarity of information, in addition to sharing course strengths and suggestions for improvement		Reduction in training time was observed. Participants liked being able to complete the training at their own pace and immediately practice the information learned *(level 1).*	
**Classroom training versus BL^f^**					
	Edwards et al [4]	Satisfaction with HIT^g^ training was assessed using a pre-existing, web-based, anonymous self-report survey. The instrument consisted of 13 questions: 9 questions focused on satisfaction with course execution, instructor quality, and usefulness of materials Ratings from these 9 questions were summed to form a single satisfaction score. Three subjective questions: identify the most and least valuable information and materials experienced during training Additional question elicited general comments and suggestions		Learners were equally satisfied with both methods: instructor-led and blended learning BL *(level 1).* Instructor-led participants found the training to be valuable *(level 1),* particularly the system functions and navigation.	
**Simulation training**					
	Vuk et al [19]	Questionnaires on a 7-point Likert scale before and immediately after simulation training Assessed their perceptions about the importance of EMRs in improving patients’ safety and their confidence and preparedness level to use EMRs		Simulation training enhanced physicians’ and nurses’ levels of self-confidence and preparedness to use EMRs *(level 2a).*	

^a^EMR: electronic medical record.

^b^EHR: electronic health record.

^c^CPOM: computerized physician order management.

^d^HIS: health information system.

^e^ICT: information computer technology.

^f^BL: blended learning.

^g^HIT: health information technology.

## Discussion

### Current State of HIS Education Programs

This study identified critical knowledge gaps in our understanding of the most effective strategies and approaches in enabling health care providers to optimally use an HIS system. We only identified 17 studies that examined the effects of different education tactics in a hospital environment. This paucity of literature indicates the maturity of the topic area and emphasizes the need to establish a baseline understanding of HIS education strategies during and post implementation. This study provides an understanding of the current landscape of these programs and important insights into successful education development and improvement.

HIS education and training have been identified as potential key facilitators in ensuring effective and optimal use of technology and can have a positive impact on HIS implementation, efficiency, and patient care. Competency in HIS is now an essential clinical skill, and health care providers and staff who lack proficiency and efficiency may face challenges in performing clinical tasks [29]. Despite this, many organizations underestimate education and training needs and the time required for effective education [10]. Unsuccessful transitions are also because of a lack of understanding of what learners expect to gain from training and failure to link training to HIS implementation. Furthermore, a lack of adequate education may increase the risk of users creating workarounds that limit the advantages of HIS and potentially hold the organization back [10].

Upon review of the numerous instruments employed in the reviewed literature, it is apparent that no standardized tools have been adopted yet to assess learner outcomes of education programs. The majority of authors used self-constructed, nonvalidated scales and defined their results in qualitative terms. This limits all future efforts to compare and analyze evidence on the effectiveness of the HIS curriculum. More studies with standardized outcome measures and assessment tools are required to support recommendations on the most effective approaches in enabling providers to optimally use an HIS.

Fortunately, HIS training is beginning to embrace a learner-centric paradigm, and HIS education can be informed by existing educational frameworks such as Kirkpatrick and Moore. For example, Kirkpatrick and Moore’s frameworks are focused on evaluating health care provider education and have become a commonly cited reference when assessing educational outcomes. Although these frameworks provide a good source of reference for evaluating the impact of learning and development, these frameworks do not provide sufficient direction for designing and sustaining an HIS education program. In particular, the frameworks do not take into account a wide array of factors, including those associated with the organization, individuals, teams, and the overall design of the education itself, all of which can influence the effectiveness of the educational program before, during, or after education delivery. Similarly, the experiential learning theory by Kolb focuses on learning at the individual level, a helpful but insufficient perspective to guide planning for HIS education across an organization. Finally, and importantly, none of these frameworks are grounded in evidence unique to HIS education.

**Figure 2:**
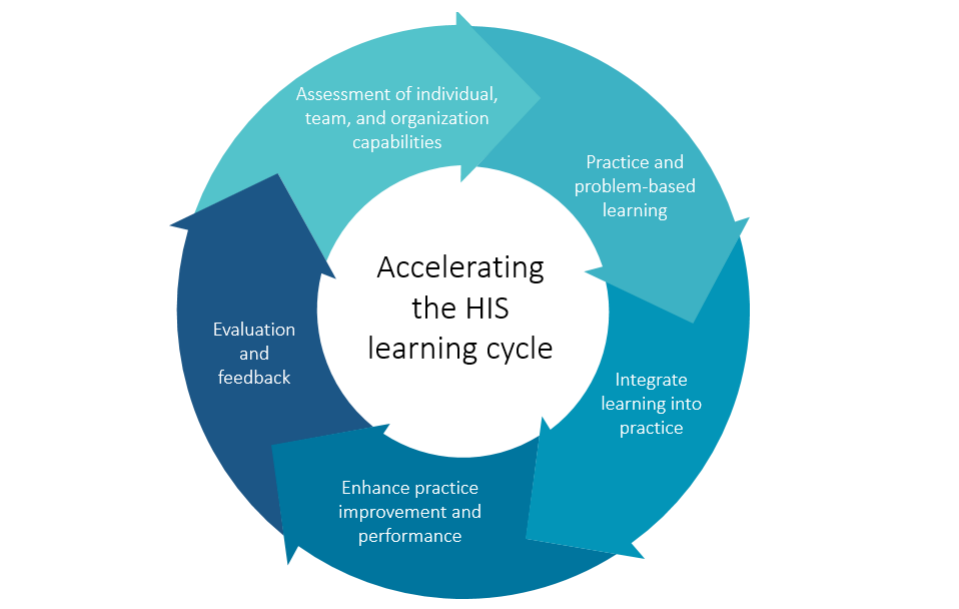
Accelerating the health information system learning cycle framework. HIS: Health Information System.

Given many of the considerations that are unique to HISs and the limited applicability of existing educational frameworks, the authors developed a new framework for HIS education that guides the adoption of the most effective education strategies used to equip health care providers with the skills required to work effectively in a clinical environment. To address these gaps, the Accelerating the HIS Learning Cycle Framework in Figure 2, with the 5 fundamental elements, was developed from the data from this study.

### Research Question 3: Most Effective Approaches in Enabling Individuals to Optimally Use an HIS

#### Assessment of Individual, Team, and Organization Capabilities

The assessment of the current capability of learners (individuals and teams) before the delivery of training appears to be a critical facet of effective HIS implementation. As learners progress in their environment and acquire new skills and methods of engaging in their ecosystem, education should be adapted to address their learning needs. Hence, it is worth acknowledging that there may be different levels of HIS knowledge and adoption among providers. Terry et al [30] and Harton et al [31] noted that although there is variability regarding the influence of previous technical knowledge on perceptions of EMR adoption, learners with lower digital literacy may require extra sessions to learn the functionality of the computer and the processes necessary for clinical practice. This finding is particularly important as health care systems evolve to leverage HISs to support clinicians’ adaptation to the new workflows and integration of this technology into their clinical practice. Despite the value placed on interprofessional learning, the literature has primarily focused on individual learning and a dearth of evidence has been provided on team-based learning. Future research exploring interprofessional education could provide greater insights into designing an effective educational program.

Culture pervades learning, and to meet the needs of diverse learners, issues revolving around the social and cultural dimensions of task design, structuring of content, and communication channels must be considered when designing a curriculum [32]. Culturally responsive pedagogy (CRP) recognizes learners’ differences and stresses that the cultural congruence of an instructional environment increases learners’ success [33]. A study from the University of British Columbia revealed that learners who are culturally diverse have a tenuous relationship with institutions that focus their curriculum on traditional, Eurocentric, and normative approaches [33]. These approaches tend to neglect learners from marginalized cultural backgrounds by disregarding their cultural habitus, leading to a cultural discontinuity for learners and the organization [33]. Rijal [34] reported that learning organizations moving toward a culture that encourages openness, creativity, experimentation, and tolerance for mistakes will enhance learning outcomes. An important part of a learning organization is being able to create new knowledge and use it to capitalize on new opportunities [35]. CRP has been hypothesized to connect all facets of learning with each other on emotional, social, mental, and physical levels [33]. In this framework, CRP is an important element in understanding individuals’ and organizations’ needs.

#### Practice and Problem-Based Learning

This study surfaced the relevance of problem-based learning in HIS education and training. A majority of the papers focused on a task-based learning approach to training, where learning is built based on predefined tasks addressed in clinical practice. Unfortunately, a task-based approach can lead to learners being dependent on instructors and support tools to provide them with guidance to perform the task, rather than encouraging them to critically think about how to approach the task [36]. Focusing on a narrow learning parameter without examining the larger context of the HIS system may not be sufficient to successfully transform learners from the existing approach to the new electronic documentation [17]. Problem-based learning provides a promising avenue for delivering an optimal learning experience while fostering an active independent learning attitude in learners. This learning approach enables learners to internalize knowledge through a process of solving clinical problems and stimulate deeper thinking with an emphasis on *how and why* questions [36]. Education that is at the appropriate skill level of learners and focused on a problem-based learning approach may encourage learners to critically reflect and attempt to understand not only the tasks themselves but also the concepts and mechanisms underlying the tasks.

This study underscores the importance of incorporating hands-on practice as part of education to increase learners’ confidence and competency in successfully using the HIS system. In a review by Younge et al [37], the authors asserted that hands-on practice addressed the learner’s level of computer literacy, which also relates to their ability regarding the ease or difficulty of using HIS. Similarly, Youssef [10] contended that hands-on experience enables learners to develop realistic expectations of what the HIS is able to offer. Thus, learners are able to strengthen the connection between personal experiences, learning content, knowledge, and a concrete task, resulting in better comprehension of abstract concepts [38].

#### Integrate Learning Into Practice

Iterative assessment of learners’ performance with new scenarios enables learners to demonstrate their knowledge and their competency in using their knowledge to deal with the new and more difficult cases being presented. Practice-based learning strengthens learners’ knowledge integration and application in a real-life setting [38]. Younge et al [37] noted that educating with materials, which provide opportunities for active learning and using assessments that evaluate what learners know (efficiency) and how they use existing knowledge to solve new problems helps to foster adaptive expertise. Learners highlighted real-life scenarios as a way to augment critical thinking by engaging in discussions. Hence, digital learning resources must be designed in a manner that offers better immersion while not increasing cognitive load [38].

Use of hands-on learning is consistent with the main phases of the experiential learning theory by Kolb [39]. The incorporation of hands-on practice and real case scenarios provides learners with an opportunity to deal with the workflows in clinical practice, which occurs when clinicians engage in an uncertain and unfamiliar context and allows learners to take an active role in the learning process. Using hands-on practice and case studies built on the 4 stages of the experiential learning theory would elicit evidence for changes in the cognitive process, learning, and behavior. This is critical in underpinning the design of an HIS curriculum and the role of educators and learners.

The clinical environment is an ideal setting to identify knowledge and skill gaps and then pursue learning with colleagues and instructors in a venture to fill these gaps. The authors advocate for the development of an interprofessional community of practice (CoP) as it facilitates the sharing of best practices and allows for the creation of new knowledge to advance the domain of HIS in clinical practice [40]. A CoP is built based on the assumptions of co-participation, where all learners from varied geographical locations engage in the activities of the community intending to facilitate meaningful learning [41]. As long as learners and instructors are present and engaged, an online community will evolve dynamically to meet their specific needs [41]. The digital space allows participants to share their experiences and knowledge in creative ways to cultivate new approaches to problems. When designing the HIS education strategy, this element of sustainability should be considered to enable individuals to optimally adopt and effectively use an HIS.

#### Enhance Practice Improvement and Performance

Another critical element as part of an educational strategy is identifying and engaging super users early in the project to help foster learning and understand the value of the HIS in clinical settings. Engaging super users in the development of an educational program enables the content to be tailored to specific provider needs, which, in turn, will contribute to the overall HIS adoption and successful implementation. The identification and engagement of champions and super users are rooted in the diffusion of innovation theory developed by Rogers [42]. Super users and champions are early adopters and innovators who adopt the HIS very early and take part in the dissemination of the new idea within the organization. They use the communication channels established to influence people’s attitudes and accelerate the rate of adoption [42].

#### Evaluation and Feedback

Evaluation plans should not only evaluate the efficacy of the initial training but also use this information to inform plans to address the ongoing learning needs during and post-HIS education and implementation. In addition, ongoing evaluation can provide insights into emerging learning needs not only about the HIS system but also about emerging practice gaps and variations, which can help refine the goals and objectives and guide the implementation of the most effective education strategies. Through evaluation associated with HIS education and implementation, areas of significant strength can also be identified; for example, one part of the organization may demonstrate exceptional use of the HIS in practice. Positive deviance allows the organization to identify the top performers and foster analysis and discussion of such performance to elevate performance among other groups within the organization [43]. This approach characterizes not only the processes and practices that exist in top-performing groups but also the context in which they are implemented, such as the organizational culture and norms of behaviors [43]. Evaluation can be used to maintain and garner support for an education program in addition to assessing learner achievement.

### Limitations

The findings of our scoping review should be examined in the context of the following limitations. Due to the nature of the scoping review, the quality of each study was not assessed. The age of the literature and the gap in publication dates may curtail the validity of the findings concerning the current landscape, as many of the previous papers were published in a different health care climate. Moreover, based on 5 studies that assessed the third level of Kirkpatrick, one was a perceived outcome in behavior, and only one of the studies assessed the highest level of Kirkpatrick-Barr (results). Given the nature of the topic being investigated, we excluded studies that discussed HIS education in academic institutions (eg, universities). Another limitation of this study is that we cannot confirm that we did not miss any relevant studies as the literature on the most effective approaches in enabling individuals to optimally use an HIS is heterogeneous. There is no standardized terminology in educational research, and the term used to describe the same ideas may vary depending on the author, thus limiting the retrieval of papers comprising important findings. The acceleration of the HIS learning cycle framework is emergent from this study and itself has not yet been validated.

### Conclusions

This study supports the development of an HIS learning framework that educators can use to guide the design and development of HIS education and training during and postimplementation. Given the advances in HISs, health care organizations should be equipped with the essential tools to deal with the turbulence that embodies digital ecosystems and research into all facets of education that prepare health providers, teams, and the organization as a whole, for the rapidly changing nature of clinical environments. This framework is a novel addition to the literature and needs to be pilot tested to evaluate their feasibility and efficacy in health care education. We posit that to successfully transform care providers to use the new technology, best practices and training principles in HIS education that harness the nature of transformative learning must be pursued. Future efforts should examine the effectiveness of interprofessional education interventions, as the literature predominantly focuses on individualized learning. Finally, we encourage future studies to focus on iterative learning to better understand how providers continue to learn from the HIS postimplementation about key practice gaps.

## Appendix

Multimedia Appendix 1 PRISMA (Preferred Reporting Items for Systematic Review and Meta-analysis) scoping review checklist.

Multimedia Appendix 2 Search strategy.

## Abbreviations

CRP: culturally responsive pedagogy
EHR: electronic health record
EMR: electronic medical record
HIS: Health Information System
PRISMA: Preferred Reporting Items for Systematic Review and Meta-Analysis
