# Supplementary material for: An Education Framework for Effective Implementation of a Health Information System: Scoping Review
Source: J Med Internet Res. 2021 Feb 24;23(2):e24691. doi: 10.2196/24691 (PMC7946593; doi:10.2196/24691)
Supplement: Multimedia Appendix 2 [file jmir_v23i2e24691_app2.docx]

Multimedia Appendix 2. Search strategy.

November 19, 2019

Database: Ovid MEDLINE(R) ALL <1946 to November 15, 2019>

Search Strategy:

--------------------------------------------------------------------------------

1 exp Medical Records Systems, Computerized/ (38672)

2 exp Hospital Information Systems/ (26352)

3 electronic health record?.ti,kf,hw. (21015)

4 electronic medical record?.ti,kf,hw. (3373)

5 electronic hospital record?.ti,kf,hw. (7)

6 electronic patient record?.ti,kf,hw. (558)

7 health information system?.ti,kf,hw. (2080)

8 computer* health record?.ti,kf,hw. (4)

9 computer* medical record?.ti,kf,hw. (240)

10 computer* hospital record?.ti,kf,hw. (0)

11 computer* patient record?.ti,kf,hw. (133)

12 hospital?? information system?.ti,kf,hw. (11229)

13 clinic?? information system?.ti,kf,hw. (502)

14 electronic health medical record?.ti,kf,hw. (0)

15 electronic hospital medical record?.ti,kf,hw. (0)

16 record? system?.ti,kf,hw. (19790)

17 (ehr or emr or epr or ehmr).ti,kf,hw. (9075)

18 or/1-17 (72012)

19 exp Teaching/ (83267)

20 Education, Distance/ (3754)

21 in-person.mp. (9509)

22 inperson.mp. (35)

23 (face-to-face adj6 train*).mp. (757)

24 (face-to-face adj6 educat*).mp. (568)

25 (face-to-face adj6 learn*).mp. (357)

26 (face-to-face adj6 teach*).mp. (223)

27 (face-to-face adj6 instruct*).mp. (148)

28 (face-to-face adj6 course?).mp. (170)

29 (face-to-face adj6 class??).mp. (76)

30 (one-on-one adj6 train*).mp. (162)

31 (one-on-one adj6 educat*).mp. (209)

32 (one-on-one adj6 learn*).mp. (26)

33 (one-on-one adj6 teach*).mp. (85)

34 (one-on-one adj6 instruct*).mp. (73)

35 (individual* adj6 train*).mp. (10529)

36 (individual* adj6 educat*).mp. (9509)

37 (individual* adj6 learn*).mp. (6998)

38 (individual* adj6 teach*).mp. (1703)

39 (individual* adj6 instruct*).mp. (1507)

40 (group? adj6 train*).mp. (31672)

41 (group? adj6 educat*).mp. (23560)

42 (group? adj6 learn*).mp. (10600)

43 (group? adj6 teach*).mp. (5459)

44 (group? adj6 instruct*).mp. (4701)

45 (session? adj6 train*).mp. (17642)

46 (session? adj6 educat*).mp. (6042)

47 (session? adj6 learn*).mp. (2852)

48 (session? adj6 teach*).mp. (2099)

49 (session? adj6 instruct*).mp. (1022)

50 classroom.mp. (14053)

51 (teach* adj4 modalit*).mp. (430)

52 (teach* adj4 format*).mp. (620)

53 (learn* adj4 modalit*).mp. (670)

54 (learn* adj4 format*).mp. (2026)

55 (train* adj4 modalit*).mp. (1346)

56 (train* adj4 format*).mp. (1043)

57 (educat* adj4 modalit*).mp. (550)

58 (educat* adj4 format*).mp. (1060)

59 (multimodal* adj6 educat*).mp. (263)

60 (multi-modal* adj6 educat*).mp. (45)

61 (multimodal* adj6 teach*).mp. (70)

62 (multi-modal* adj6 teach*).mp. (13)

63 (multimodal* adj6 learn*).mp. (387)

64 (multi-modal* adj6 learn*).mp. (105)

65 (multimodal* adj6 instruct*).mp. (21)

66 (multi-modal* adj6 instruct*).mp. (5)

67 (multimodal* adj6 train*).mp. (346)

68 (multi-modal* adj6 train*).mp. (70)

69 (combin* adj4 modalit* adj6 educat*).mp. (220)

70 (combin* adj4 modalit* adj6 teach*).mp. (7)

71 (combin* adj4 modalit* adj6 learn*).mp. (28)

72 (combin* adj4 modalit* adj6 instruct*).mp. (5)

73 (combin* adj4 modalit* adj6 train*).mp. (67)

74 (teach* adj6 material?).mp. (8433)

75 (educat* adj6 material?).mp. (7945)

76 (train* adj6 material?).mp. (3342)

77 (online adj6 train*).mp. (1899)

78 (online adj6 learn*).mp. (2912)

79 (online adj6 teach*).mp. (814)

80 (online adj6 instruct*).mp. (1585)

81 (online adj6 course).mp. (1140)

82 (online adj6 class??).mp. (356)

83 e?learn*.mp. (251)

84 (online adj6 educat*).mp. (2842)

85 (portal adj6 train*).mp. (66)

86 (portal adj6 learn*).mp. (64)

87 (portal adj6 teach*).mp. (18)

88 (portal adj6 instruct*).mp. (9)

89 (portal adj6 educat*).mp. (136)

90 webinar*.mp. (496)

91 workshop*.mp. (38029)

92 tutorial?.mp. (7119)

93 instructor*.mp. (8050)

94 (web-based adj6 educat*).mp. (1127)

95 (web-based adj6 train*).mp. (716)

96 (web-based adj6 learn*).mp. (840)

97 (web-based adj6 teach*).mp. (390)

98 (web-based adj6 instruct*).mp. (224)

99 (web-based adj6 course*).mp. (369)

100 (web-based adj6 class??).mp. (50)

101 (distance adj6 educat*).mp. (4623)

102 (distance adj6 train*).mp. (1659)

103 (distance adj6 learn*).mp. (2152)

104 (distance adj6 teach*).mp. (215)

105 (distance adj6 instruct*).mp. (511)

106 (remote adj6 educat*).mp. (388)

107 (remote adj6 train*).mp. (463)

108 (remote adj6 learn*).mp. (296)

109 (remote adj6 teach*).mp. (105)

110 (remote adj6 instruct*).mp. (64)

111 (module* adj6 educat*).mp. (1529)

112 (module* adj6 train*).mp. (1752)

113 (module* adj6 learn*).mp. (1956)

114 (module* adj6 teach*).mp. (905)

115 (module* adj6 instruct*).mp. (347)

116 (application* adj6 educat*).mp. (2998)

117 (application* adj6 train*).mp. (3380)

118 (application* adj6 learn*).mp. (4125)

119 (application* adj6 teach*).mp. (1026)

120 (application* adj6 instruct*).mp. (679)

121 (video* adj6 educat*).mp. (2627)

122 (video* adj6 train*).mp. (2840)

123 (video* adj6 learn*).mp. (1509)

124 (video* adj6 teach*).mp. (2244)

125 (video* adj6 instruct*).mp. (1436)

126 (video* adj6 course*).mp. (466)

127 (video* adj6 class??).mp. (356)

128 didactic.mp. (7003)

129 (lecture? or lecturing).mp. (33870)

130 hands-on.mp. (6197)

131 peer-to-peer.mp. (1240)

132 problem-based.mp. (10324)

133 (interactive adj6 educat*).mp. (1769)

134 (interactive adj6 train*).mp. (1088)

135 (interactive adj6 learn*).mp. (1747)

136 (interactive adj6 teach*).mp. (1033)

137 (interactive adj6 instruct*).mp. (381)

138 (interactive adj6 course*).mp. (408)

139 (interactive adj6 class??).mp. (181)

140 (real-world adj6 educat*).mp. (100)

141 (real-world adj6 train*).mp. (265)

142 (real-world adj6 learn*).mp. (422)

143 (real-world adj6 teach*).mp. (59)

144 (real-world adj6 instruct*).mp. (32)

145 simulat*.mp. (620358)

146 realistic environment*.mp. (463)

147 (learn* adj6 environment*).mp. (9524)

148 (teach* adj6 environment*).mp. (2067)

149 (educat* adj6 environment*).mp. (8095)

150 (instruct* adj6 environment*).mp. (522)

151 (blended adj6 train*).mp. (120)

152 (blended adj6 learn*).mp. (732)

153 (blended adj6 educat*).mp. (179)

154 (blended adj6 teach*).mp. (125)

155 (blended adj6 instruct*).mp. (33)

156 (blended adj6 course?).mp. (156)

157 (blended adj6 class??).mp. (24)

158 (blended adj6 modalit*).mp. (10)

159 (hybrid adj6 train*).mp. (371)

160 (hybrid adj6 learn*).mp. (547)

161 (hybrid adj6 educat*).mp. (98)

162 (hybrid adj6 teach*).mp. (63)

163 (hybrid adj6 instruct*).mp. (61)

164 (hybrid adj6 course?).mp. (156)

165 (hybrid adj6 class??).mp. (1123)

166 (hybrid* adj6 modalit*).mp. (392)

167 or/19-166 (949399)

168 18 and 167 (3993)

169 implement*.mp. (460082)

170 inaugurat*.mp. (1357)

171 (tech* adj4 adopt*).mp. (8505)

172 (system* adj4 adopt*).mp. (5503)

173 (ehr adj4 adopt*).mp. (409)

174 (emr adj4 adopt*).mp. (149)

175 (epr adj4 adopt*).mp. (23)

176 (ehmr adj4 adopt*).mp. (0)

177 uptake.mp. (372177)

178 rollout.mp. (885)

179 launch???.mp. (25019)

180 or/169-179 (860278)

181 168 and 180 (960)

***************************

November 19, 2019

Database: Embase Classic+Embase <1947 to 2019 November 15>

Search Strategy:

--------------------------------------------------------------------------------

1 electronic medical record/ (52056)

2 electronic health record/ (14135)

3 electronic patient record/ (1967)

4 medical information system/ (20376)

5 nursing information system/ (74)

6 electronic health record?.ti,kw,hw. (18639)

7 electronic medical record?.ti,kw,hw. (54491)

8 electronic hospital record?.ti,kw,hw. (9)

9 electronic patient record?.ti,kw,hw. (2762)

10 health information system?.ti,kw,hw. (1378)

11 computer* health record?.ti,kw,hw. (3)

12 computer* medical record?.ti,kw,hw. (421)

13 computer* hospital record?.ti,kw,hw. (0)

14 computer* patient record?.ti,kw,hw. (215)

15 hospital?? information system?.ti,kw,hw. (20633)

16 clinic?? information system?.ti,kw,hw. (668)

17 electronic health medical record?.ti,kw,hw. (0)

18 electronic hospital medical record?.ti,kw,hw. (0)

19 record? system?.ti,kw,hw. (2805)

20 (ehr or emr or epr or ehmr).ti,kw,hw. (12685)

21 or/1-20 (119134)

22 exp teaching/ (97387)

23 education/ (427175)

24 educational model/ (8118)

25 educational technology/ (3002)

26 in service training/ (16156)

27 in-person.mp. (14482)

28 inperson.mp. (348)

29 (face-to-face adj6 train*).mp. (1221)

30 (face-to-face adj6 educat*).mp. (883)

31 (face-to-face adj6 learn*).mp. (531)

32 (face-to-face adj6 teach*).mp. (357)

33 (face-to-face adj6 instruct*).mp. (198)

34 (face-to-face adj6 course?).mp. (258)

35 (face-to-face adj6 class??).mp. (103)

36 (one-on-one adj6 train*).mp. (312)

37 (one-on-one adj6 educat*).mp. (379)

38 (one-on-one adj6 learn*).mp. (59)

39 (one-on-one adj6 teach*).mp. (158)

40 (one-on-one adj6 instruct*).mp. (109)

41 (individual* adj6 train*).mp. (14840)

42 (individual* adj6 educat*).mp. (13597)

43 (individual* adj6 learn*).mp. (9219)

44 (individual* adj6 teach*).mp. (2401)

45 (individual* adj6 instruct*).mp. (2036)

46 (group? adj6 train*).mp. (44639)

47 (group? adj6 educat*).mp. (36605)

48 (group? adj6 learn*).mp. (14928)

49 (group? adj6 teach*).mp. (7704)

50 (group? adj6 instruct*).mp. (6338)

51 (session? adj6 train*).mp. (26000)

52 (session? adj6 educat*).mp. (11244)

53 (session? adj6 learn*).mp. (4814)

54 (session? adj6 teach*).mp. (4043)

55 (session? adj6 instruct*).mp. (1622)

56 classroom.mp. (17198)

57 (teach* adj4 modalit*).mp. (691)

58 (teach* adj4 format*).mp. (879)

59 (learn* adj4 modalit*).mp. (943)

60 (learn* adj4 format*).mp. (2709)

61 (train* adj4 modalit*).mp. (1865)

62 (train* adj4 format*).mp. (1460)

63 (educat* adj4 modalit*).mp. (896)

64 (educat* adj4 format*).mp. (1558)

65 (multimodal* adj6 educat*).mp. (451)

66 (multi-modal* adj6 educat*).mp. (121)

67 (multimodal* adj6 teach*).mp. (108)

68 (multi-modal* adj6 teach*).mp. (34)

69 (multimodal* adj6 learn*).mp. (535)

70 (multi-modal* adj6 learn*).mp. (167)

71 (multimodal* adj6 instruct*).mp. (39)

72 (multi-modal* adj6 instruct*).mp. (8)

73 (multimodal* adj6 train*).mp. (486)

74 (multi-modal* adj6 train*).mp. (135)

75 (combin* adj4 modalit* adj6 educat*).mp. (17)

76 (combin* adj4 modalit* adj6 teach*).mp. (12)

77 (combin* adj4 modalit* adj6 learn*).mp. (26)

78 (combin* adj4 modalit* adj6 instruct*).mp. (6)

79 (combin* adj4 modalit* adj6 train*).mp. (63)

80 (teach* adj6 material?).mp. (3968)

81 (educat* adj6 material?).mp. (12657)

82 (train* adj6 material?).mp. (5740)

83 (online adj6 train*).mp. (3328)

84 (online adj6 learn*).mp. (4338)

85 (online adj6 teach*).mp. (1244)

86 (online adj6 instruct*).mp. (1711)

87 (online adj6 course).mp. (1720)

88 (online adj6 class??).mp. (491)

89 e?learn*.mp. (626)

90 (online adj6 educat*).mp. (5013)

91 (portal adj6 train*).mp. (138)

92 (portal adj6 learn*).mp. (158)

93 (portal adj6 teach*).mp. (35)

94 (portal adj6 instruct*).mp. (15)

95 (portal adj6 educat*).mp. (247)

96 webinar*.mp. (1145)

97 workshop*.mp. (72287)

98 tutorial?.mp. (7588)

99 instructor*.mp. (10764)

100 (web-based adj6 educat*).mp. (1697)

101 (web-based adj6 train*).mp. (1181)

102 (web-based adj6 learn*).mp. (1182)

103 (web-based adj6 teach*).mp. (523)

104 (web-based adj6 instruct*).mp. (291)

105 (web-based adj6 course*).mp. (463)

106 (web-based adj6 class??).mp. (67)

107 (distance adj6 educat*).mp. (2039)

108 (distance adj6 train*).mp. (2109)

109 (distance adj6 learn*).mp. (2596)

110 (distance adj6 teach*).mp. (269)

111 (distance adj6 instruct*).mp. (192)

112 (remote adj6 educat*).mp. (558)

113 (remote adj6 train*).mp. (699)

114 (remote adj6 learn*).mp. (378)

115 (remote adj6 teach*).mp. (133)

116 (remote adj6 instruct*).mp. (91)

117 (module* adj6 educat*).mp. (2707)

118 (module* adj6 train*).mp. (3264)

119 (module* adj6 learn*).mp. (3323)

120 (module* adj6 teach*).mp. (1459)

121 (module* adj6 instruct*).mp. (553)

122 (application* adj6 educat*).mp. (4046)

123 (application* adj6 train*).mp. (4738)

124 (application* adj6 learn*).mp. (5169)

125 (application* adj6 teach*).mp. (1433)

126 (application* adj6 instruct*).mp. (908)

127 (video* adj6 educat*).mp. (4646)

128 (video* adj6 train*).mp. (5347)

129 (video* adj6 learn*).mp. (2547)

130 (video* adj6 teach*).mp. (3643)

131 (video* adj6 instruct*).mp. (2261)

132 (video* adj6 course*).mp. (818)

133 (video* adj6 class??).mp. (519)

134 didactic.mp. (12057)

135 (lecture? or lecturing).mp. (40459)

136 hands-on.mp. (10690)

137 peer-to-peer.mp. (1819)

138 problem-based.mp. (10126)

139 (interactive adj6 educat*).mp. (2846)

140 (interactive adj6 train*).mp. (1774)

141 (interactive adj6 learn*).mp. (2785)

142 (interactive adj6 teach*).mp. (1581)

143 (interactive adj6 instruct*).mp. (523)

144 (interactive adj6 course*).mp. (657)

145 (interactive adj6 class??).mp. (281)

146 (real-world adj6 educat*).mp. (136)

147 (real-world adj6 train*).mp. (339)

148 (real-world adj6 learn*).mp. (496)

149 (real-world adj6 teach*).mp. (73)

150 (real-world adj6 instruct*).mp. (34)

151 simulat*.mp. (612811)

152 realistic environment*.mp. (512)

153 (learn* adj6 environment*).mp. (13464)

154 (teach* adj6 environment*).mp. (3031)

155 (educat* adj6 environment*).mp. (16510)

156 (instruct* adj6 environment*).mp. (679)

157 (blended adj6 train*).mp. (185)

158 (blended adj6 learn*).mp. (1063)

159 (blended adj6 educat*).mp. (213)

160 (blended adj6 teach*).mp. (182)

161 (blended adj6 instruct*).mp. (47)

162 (blended adj6 course?).mp. (237)

163 (blended adj6 class??).mp. (32)

164 (blended adj6 modalit*).mp. (13)

165 (hybrid adj6 train*).mp. (500)

166 (hybrid adj6 learn*).mp. (736)

167 (hybrid adj6 educat*).mp. (139)

168 (hybrid adj6 teach*).mp. (91)

169 (hybrid adj6 instruct*).mp. (73)

170 (hybrid adj6 course?).mp. (208)

171 (hybrid adj6 class??).mp. (1122)

172 (hybrid* adj6 modalit*).mp. (592)

173 or/22-172 (1419903)

174 21 and 173 (8805)

175 implement*.mp. (606140)

176 inaugurat*.mp. (1928)

177 (tech* adj4 adopt*).mp. (11767)

178 (system* adj4 adopt*).mp. (6944)

179 (ehr adj4 adopt*).mp. (445)

180 (emr adj4 adopt*).mp. (195)

181 (epr adj4 adopt*).mp. (18)

182 (ehmr adj4 adopt*).mp. (0)

183 uptake.mp. (523149)

184 rollout.mp. (1344)

185 launch???.mp. (36857)

186 or/175-185 (1167057)

187 174 and 186 (2629)

188 limit 187 to conference abstract status (1317)

189 187 not 188 (1312)

***************************

| Friday, November 22, 2019 12:54:46 PM  Interface - EBSCOhost Research Databases Search Screen - Advanced Search Database - CINAHL with Full Text |
| --- |

| **#** | **Query** | **Limiters/Expanders** | **Results** |
| --- | --- | --- | --- |
| S179 | S165 AND S177 | Limiters - Publication Type: Journal Article Search modes - Boolean/Phrase | 1,919 |
| S178 | S165 AND S177 | Search modes - Boolean/Phrase | 2,716 |
| S177 | S166 OR S167 OR S168 OR S169 OR S170 OR S171 OR S172 OR S173 OR S174 OR S175 OR S176 | Search modes - Boolean/Phrase | 228,872 |
| S176 | launch* | Search modes - Boolean/Phrase | 16,649 |
| S175 | rollout | Search modes - Boolean/Phrase | 562 |
| S174 | uptake | Search modes - Boolean/Phrase | 35,861 |
| S173 | ehmr N4 adopt* | Search modes - Boolean/Phrase | 0 |
| S172 | epr N4 adopt* | Search modes - Boolean/Phrase | 7 |
| S171 | emr N4 adopt* | Search modes - Boolean/Phrase | 117 |
| S170 | ehr N4 adopt* | Search modes - Boolean/Phrase | 477 |
| S169 | system* N4 adopt* | Search modes - Boolean/Phrase | 1,918 |
| S168 | tech* N4 adopt* | Search modes - Boolean/Phrase | 2,748 |
| S167 | inaugurat* | Search modes - Boolean/Phrase | 280 |
| S166 | implement* | Search modes - Boolean/Phrase | 176,544 |
| S165 | S17 AND S164 | Search modes - Boolean/Phrase | 8,019 |
| S164 | S18 OR S19 OR S20 OR S21 OR S22 OR S23 OR S24 OR S25 OR S26 OR S27 OR S28 OR S29 OR S30 OR S31 OR S32 OR S33 OR S34 OR S35 OR S36 OR S37 OR S38 OR S39 OR S40 OR S41 OR S42 OR S43 OR S44 OR S45 OR S46 OR S47 OR S48 OR S49 OR S50 OR S51 OR S52 OR S53 OR S54 OR S55 OR S56 OR S57 OR S58 OR S59 OR S60 OR S61 OR S62 OR S63 OR S64 OR S65 OR S66 OR S67 OR S68 OR S69 OR S70 OR S71 OR S72 OR S73 OR S74 OR S75 OR S76 OR S77 OR S78 OR S79 OR S80 OR S81 OR S82 OR S83 OR S84 OR S85 OR S86 OR S87 OR S88 OR S89 OR S90 OR S91 OR S92 OR S93 OR S94 OR S95 OR S96 OR S97 OR S98 OR S99 OR S100 OR S101 OR S102 OR S103 OR S104 OR S105 OR S106 OR S107 OR S108 OR S109 OR S110 OR S111 OR S112 OR S113 OR S114 OR S115 OR S116 OR S117 OR S118 OR S119 OR S120 OR S121 OR S122 OR S123 OR S124 OR S125 OR S126 OR S127 OR S128 OR S129 OR S130 OR S131 OR S132 OR S133 OR S134 OR S135 OR S136 OR S137 OR S138 OR S139 OR S140 OR S141 OR S142 OR S143 OR S144 OR S145 OR S146 OR S147 OR S148 OR S149 OR S150 OR S151 OR S152 OR S153 OR S154 OR S155 OR S156 OR S157 OR S158 OR S159 OR S160 OR S161 OR S162 OR S163 | Search modes - Boolean/Phrase | 841,200 |
| S163 | TI (hybrid* N6 modalit*) OR SU (hybrid* N6 modalit*) | Search modes - Boolean/Phrase | 6 |
| S162 | TI (hybrid N6 class*) OR SU (hybrid N6 class*) | Search modes - Boolean/Phrase | 42 |
| S161 | TI (hybrid N6 course*) OR SU (hybrid N6 course*) | Search modes - Boolean/Phrase | 27 |
| S160 | TI (hybrid N6 instruct*) OR SU (hybrid N6 instruct*) | Search modes - Boolean/Phrase | 9 |
| S159 | TI (hybrid N6 teach*) OR SU (hybrid N6 teach*) | Search modes - Boolean/Phrase | 18 |
| S158 | TI (hybrid N6 educat*) OR SU (hybrid N6 educat*) | Search modes - Boolean/Phrase | 41 |
| S157 | TI (hybrid N6 learn*) OR SU (hybrid N6 learn*) | Search modes - Boolean/Phrase | 43 |
| S156 | TI (hybrid N6 train*) OR SU (hybrid N6 train*) | Search modes - Boolean/Phrase | 65 |
| S155 | TI (blended N6 modalit*) OR SU (blended N6 modalit*) | Search modes - Boolean/Phrase | 1 |
| S154 | TI (blended N6 class*) OR SU (blended N6 class*) | Search modes - Boolean/Phrase | 12 |
| S153 | TI (blended N6 course*) OR SU (blended N6 course*) | Search modes - Boolean/Phrase | 47 |
| S152 | TI (blended N6 instruct*) OR SU (blended N6 instruct*) | Search modes - Boolean/Phrase | 7 |
| S151 | TI (blended N6 teach*) OR SU (blended N6 teach*) | Search modes - Boolean/Phrase | 36 |
| S150 | TI (blended N6 educat*) OR SU (blended N6 educat*) | Search modes - Boolean/Phrase | 39 |
| S149 | TI (blended N6 learn*) OR SU (blended N6 learn*) | Search modes - Boolean/Phrase | 263 |
| S148 | TI (blended N6 train*) OR SU (blended N6 train*) | Search modes - Boolean/Phrase | 32 |
| S147 | TI (instruct* N6 environment*) OR SU (instruct* N6 environment*) | Search modes - Boolean/Phrase | 27 |
| S146 | TI (educat* N6 environment*) OR SU (educat* N6 environment*) | Search modes - Boolean/Phrase | 1,020 |
| S145 | TI (teach* N6 environment*) OR SU (teach* N6 environment*) | Search modes - Boolean/Phrase | 208 |
| S144 | TI (learn* N6 environment*) OR SU (learn* N6 environment*) | Search modes - Boolean/Phrase | 10,194 |
| S143 | TI realistic environment* OR SU realistic environment* | Search modes - Boolean/Phrase | 2 |
| S142 | TI simulat* OR SU simulat* | Search modes - Boolean/Phrase | 37,058 |
| S141 | TI (real-world N6 instruct*) OR SU (real-world N6 instruct*) | Search modes - Boolean/Phrase | 1 |
| S140 | TI (real-world N6 teach*) OR SU (real-world N6 teach*) | Search modes - Boolean/Phrase | 14 |
| S139 | TI (real-world N6 learn*) OR SU (real-world N6 learn*) | Search modes - Boolean/Phrase | 66 |
| S138 | TI (real-world N6 train*) OR SU (real-world N6 train*) | Search modes - Boolean/Phrase | 33 |
| S137 | TI (real-world N6 educat*) OR SU (real-world N6 educat*) | Search modes - Boolean/Phrase | 33 |
| S136 | TI (interactive N6 class*) OR SU (interactive N6 class*) | Search modes - Boolean/Phrase | 26 |
| S135 | TI (interactive N6 course*) OR SU (interactive N6 course*) | Search modes - Boolean/Phrase | 40 |
| S134 | TI (interactive N6 instruct*) OR SU (interactive N6 instruct*) | Search modes - Boolean/Phrase | 78 |
| S133 | TI (interactive N6 teach*) OR SU (interactive N6 teach*) | Search modes - Boolean/Phrase | 131 |
| S132 | TI (interactive N6 learn*) OR SU (interactive N6 learn*) | Search modes - Boolean/Phrase | 247 |
| S131 | TI (interactive N6 train*) OR SU (interactive N6 train*) | Search modes - Boolean/Phrase | 136 |
| S130 | TI (interactive N6 educat*) OR SU (interactive N6 educat*) | Search modes - Boolean/Phrase | 290 |
| S129 | TI problem-based OR SU problem-based | Search modes - Boolean/Phrase | 3,095 |
| S128 | TI peer-to-peer OR SU peer-to-peer | Search modes - Boolean/Phrase | 234 |
| S127 | TI "hands on" OR SU "hands on" | Search modes - Boolean/Phrase | 4,193 |
| S126 | TI (lecture* or lecturing) OR SU (lecture* or lecturing) | Search modes - Boolean/Phrase | 5,088 |
| S125 | TI didactic OR SU didactic | Search modes - Boolean/Phrase | 271 |
| S124 | TI (video* N6 class*) OR SU (video* N6 class*) | Search modes - Boolean/Phrase | 106 |
| S123 | TI (video* N6 course*) OR SU (video* N6 course*) | Search modes - Boolean/Phrase | 30 |
| S122 | TI (video* N6 instruct*) OR SU (video* N6 instruct*) | Search modes - Boolean/Phrase | 227 |
| S121 | TI (video* N6 teach*) OR SU (video* N6 teach*) | Search modes - Boolean/Phrase | 490 |
| S120 | TI (video* N6 learn*) OR SU (video* N6 learn*) | Search modes - Boolean/Phrase | 268 |
| S119 | TI (video* N6 train*) OR SU (video* N6 train*) | Search modes - Boolean/Phrase | 348 |
| S118 | TI (video* N6 educat*) OR SU (video* N6 educat*) | Search modes - Boolean/Phrase | 607 |
| S117 | TI (application* N6 instruct*) OR SU (application* N6 instruct*) | Search modes - Boolean/Phrase | 43 |
| S116 | TI (application* N6 teach*) OR SU (application* N6 teach*) | Search modes - Boolean/Phrase | 255 |
| S115 | TI (application* N6 learn*) OR SU (application* N6 learn*) | Search modes - Boolean/Phrase | 391 |
| S114 | TI (application* N6 train*) OR SU (application* N6 train*) | Search modes - Boolean/Phrase | 337 |
| S113 | TI (application* N6 educat*) OR SU (application* N6 educat*) | Search modes - Boolean/Phrase | 597 |
| S112 | TI (module* N6 instruct*) OR SU (module* N6 instruct*) | Search modes - Boolean/Phrase | 64 |
| S111 | TI (module* N6 teach*) OR SU (module* N6 teach*) | Search modes - Boolean/Phrase | 98 |
| S110 | TI (module* N6 learn*) OR SU (module* N6 learn*) | Search modes - Boolean/Phrase | 280 |
| S109 | TI (module* N6 train*) OR SU (module* N6 train*) | Search modes - Boolean/Phrase | 157 |
| S108 | TI (module* N6 educat*) OR SU (module* N6 educat*) | Search modes - Boolean/Phrase | 316 |
| S107 | TI (remote N6 instruct*) OR SU (remote N6 instruct*) | Search modes - Boolean/Phrase | 10 |
| S106 | TI (remote N6 teach*) OR SU (remote N6 teach*) | Search modes - Boolean/Phrase | 16 |
| S105 | TI (remote N6 learn*) OR SU (remote N6 learn*) | Search modes - Boolean/Phrase | 34 |
| S104 | TI (remote N6 train*) OR SU (remote N6 train*) | Search modes - Boolean/Phrase | 70 |
| S103 | TI (remote N6 educat*) OR SU (remote N6 educat*) | Search modes - Boolean/Phrase | 90 |
| S102 | TI (distance N6 instruct*) OR SU (distance N6 instruct*) | Search modes - Boolean/Phrase | 34 |
| S101 | TI (distance N6 teach*) OR SU (distance N6 teach*) | Search modes - Boolean/Phrase | 54 |
| S100 | TI (distance N6 learn*) OR SU (distance N6 learn*) | Search modes - Boolean/Phrase | 728 |
| S99 | TI(distance N6 train*) OR SU (distance N6 train*) | Search modes - Boolean/Phrase | 141 |
| S98 | TI(distance N6 educat*) OR SU (distance N6 educat*) | Search modes - Boolean/Phrase | 722 |
| S97 | TI (web-based N6 class*) OR SU (web-based N6 class*) | Search modes - Boolean/Phrase | 16 |
| S96 | TI (web-based N6 course*) OR SU (web-based N6 course*) | Search modes - Boolean/Phrase | 111 |
| S95 | TI (web-based N6 instruct*) OR SU (web-based N6 instruct*) | Search modes - Boolean/Phrase | 61 |
| S94 | TI (web-based N6 teach*) OR SU (web-based N6 teach*) | Search modes - Boolean/Phrase | 81 |
| S93 | TI (web-based N6 learn*) OR SU (web-based N6 learn*) | Search modes - Boolean/Phrase | 200 |
| S92 | TI (web-based N6 train*) OR SU (web-based N6 train*) | Search modes - Boolean/Phrase | 174 |
| S91 | TI (web-based N6 educat*) OR SU (web-based N6 educat*) | Search modes - Boolean/Phrase | 350 |
| S90 | TI instructor* OR SU instructor* | Search modes - Boolean/Phrase | 1,620 |
| S89 | TI tutorial* OR SU tutorial* | Search modes - Boolean/Phrase | 700 |
| S88 | TI workshop* OR SU workshop* | Search modes - Boolean/Phrase | 17,932 |
| S87 | TI webinar* OR SU webinar* | Search modes - Boolean/Phrase | 1,030 |
| S86 | TI (portal N6 educat*) OR SU (portal N6 educat*) | Search modes - Boolean/Phrase | 29 |
| S85 | TI (portal N6 instruct*) OR SU (portal N6 instruct*) | Search modes - Boolean/Phrase | 2 |
| S84 | TI (portal N6 teach*) OR SU (portal N6 teach*) | Search modes - Boolean/Phrase | 1 |
| S83 | TI (portal N6 learn*) OR SU (portal N6 learn*) | Search modes - Boolean/Phrase | 13 |
| S82 | TI (portal N6 train*) OR SU (portal N6 train*) | Search modes - Boolean/Phrase | 2 |
| S81 | TI (online N6 educat*) OR SU (online N6 educat*) | Search modes - Boolean/Phrase | 1,073 |
| S80 | TI (e-learn* or elearn*) OR SU (e-learn* or elearn*) | Search modes - Boolean/Phrase | 1,383 |
| S79 | TI (online N6 class*) OR SU (online N6 class*) | Search modes - Boolean/Phrase | 155 |
| S78 | TI (online N6 course) OR SU (online N6 course) | Search modes - Boolean/Phrase | 412 |
| S77 | TI (online N6 instruct*) OR SU (online N6 instruct*) | Search modes - Boolean/Phrase | 116 |
| S76 | TI (online N6 teach*) OR SU (online N6 teach*) | Search modes - Boolean/Phrase | 278 |
| S75 | TI (online N6 learn*) OR SU (online N6 learn*) | Search modes - Boolean/Phrase | 873 |
| S74 | TI (online N6 train*) OR SU (online N6 train*) | Search modes - Boolean/Phrase | 448 |
| S73 | TI (train* N6 material*) OR SU (train* N6 material*) | Search modes - Boolean/Phrase | 75 |
| S72 | TI (educat* N6 material*) OR SU (educat* N6 material*) | Search modes - Boolean/Phrase | 807 |
| S71 | TI (teach* N6 material*) OR SU (teach* N6 material*) | Search modes - Boolean/Phrase | 10,130 |
| S70 | TI (combin* N4 modalit* N6 train*) OR SU (combin* N4 modalit* N6 train*) | Search modes - Boolean/Phrase | 4 |
| S69 | TI (combin* N4 modalit* N6 instruct*) OR SU (combin* N4 modalit* N6 instruct*) | Search modes - Boolean/Phrase | 0 |
| S68 | TI (combin* N4 modalit* N6 learn*) OR SU (combin* N4 modalit* N6 learn*) | Search modes - Boolean/Phrase | 3 |
| S67 | TI (combin* N4 modalit* N6 teach*) OR SU (combin* N4 modalit* N6 teach*) | Search modes - Boolean/Phrase | 3 |
| S66 | TI (combin* N4 modalit* N6 educat*) OR SU (combin* N4 modalit* N6 educat*) | Search modes - Boolean/Phrase | 0 |
| S65 | TI (multi-modal* N6 train*) OR SU (multi-modal* N6 train*) | Search modes - Boolean/Phrase | 14 |
| S64 | TI (multimodal* N6 train*) OR SU (multimodal* N6 train*) | Search modes - Boolean/Phrase | 46 |
| S63 | TI (multi-modal* N6 instruct*) OR SU (multi-modal* N6 instruct*) | Search modes - Boolean/Phrase | 3 |
| S62 | TI (multimodal* N6 instruct*) OR SU (multimodal* N6 instruct*) | Search modes - Boolean/Phrase | 4 |
| S61 | TI (multi-modal* N6 learn*) OR SU (multi-modal* N6 learn*) | Search modes - Boolean/Phrase | 3 |
| S60 | TI (multimodal* N6 learn*) OR SU (multimodal* N6 learn*) | Search modes - Boolean/Phrase | 28 |
| S59 | TI (multi-modal* N6 teach*) OR SU (multi-modal* N6 teach*) | Search modes - Boolean/Phrase | 4 |
| S58 | TI (multimodal* N6 teach*) OR SU (multimodal* N6 teach*) | Search modes - Boolean/Phrase | 14 |
| S57 | TI (multi-modal* N6 educat*) OR SU (multi-modal* N6 educat*) | Search modes - Boolean/Phrase | 8 |
| S56 | TI (multimodal* N6 educat*) OR SU (multimodal* N6 educat*) | Search modes - Boolean/Phrase | 21 |
| S55 | TI (educat* N4 format*) OR SU (educat* N4 format*) | Search modes - Boolean/Phrase | 111 |
| S54 | TI (educat* N4 modalit*) OR SU (educat* N4 modalit*) | Search modes - Boolean/Phrase | 36 |
| S53 | TI (train* N4 format*) OR SU (train* N4 format*) | Search modes - Boolean/Phrase | 55 |
| S52 | TI (train* N4 modalit*) OR SU (train* N4 modalit*) | Search modes - Boolean/Phrase | 82 |
| S51 | TI (learn* N4 format*) OR SU (learn* N4 format*) | Search modes - Boolean/Phrase | 95 |
| S50 | TI (learn* N4 modalit*) OR SU (learn* N4 modalit*) | Search modes - Boolean/Phrase | 29 |
| S49 | TI (teach* N4 format*) OR SU (teach* N4 format*) | Search modes - Boolean/Phrase | 62 |
| S48 | TI (teach* N4 modalit*) OR SU (teach* N4 modalit*) | Search modes - Boolean/Phrase | 16 |
| S47 | TI classroom OR SU classroom | Search modes - Boolean/Phrase | 2,968 |
| S46 | TI (session* N6 instruct*) OR SU (session* N6 instruct*) | Search modes - Boolean/Phrase | 22 |
| S45 | TI (session* N6 teach*) OR SU (session* N6 teach*) | Search modes - Boolean/Phrase | 85 |
| S44 | Ti (session* N6 learn*) OR SU (session* N6 learn*) | Search modes - Boolean/Phrase | 121 |
| S43 | TI (session* N6 educat*) OR SU (session* N6 educat*) | Search modes - Boolean/Phrase | 349 |
| S42 | TI (session* N6 train*) OR SU (session* N6 train*) | Search modes - Boolean/Phrase | 340 |
| S41 | TI (group* N6 instruct*) OR SU (group* N6 instruct*) | Search modes - Boolean/Phrase | 74 |
| S40 | TI (group* N6 teach*) OR SU (group* N6 teach*) | Search modes - Boolean/Phrase | 273 |
| S39 | TI (group* N6 learn*) OR SU (group* N6 learn*) | Search modes - Boolean/Phrase | 566 |
| S38 | TI (group* N6 educat*) OR SU (group* N6 educat*) | Search modes - Boolean/Phrase | 1,442 |
| S37 | TI (group* N6 train*) OR SU (group* N6 train*) | Search modes - Boolean/Phrase | 558 |
| S36 | TI (individual* N6 instruct*) OR SU (individual* N6 instruct*) | Search modes - Boolean/Phrase | 73 |
| S35 | TI (individual* N6 teach*) OR SU (individual* N6 teach*) | Search modes - Boolean/Phrase | 109 |
| S34 | TI (individual* N6 learn*) OR SU (individual* N6 learn*) | Search modes - Boolean/Phrase | 337 |
| S33 | TI (individual* N6 educat*) OR SU (individual* N6 educat*) | Search modes - Boolean/Phrase | 1,465 |
| S32 | TI (individual* N6 train*) OR SU (individual* N6 train*) | Search modes - Boolean/Phrase | 695 |
| S31 | TI (one-on-one N6 instruct*) OR SU (one-on-one N6 instruct*) | Search modes - Boolean/Phrase | 6 |
| S30 | TI (one-on-one N6 teach*) OR SU (one-on-one N6 teach*) | Search modes - Boolean/Phrase | 75 |
| S29 | TI (one-on-one N6 learn*) OR SU (one-on-one N6 learn*) | Search modes - Boolean/Phrase | 10 |
| S28 | TI (one-on-one N6 educat*) OR SU (one-on-one N6 educat*) | Search modes - Boolean/Phrase | 23 |
| S27 | TI (one-on-one N6 train*) OR SU (one-on-one N6 train*) | Search modes - Boolean/Phrase | 33 |
| S26 | TI (face-to-face N6 class*) OR SU (face-to-face N6 class*) | Search modes - Boolean/Phrase | 9 |
| S25 | TI (face-to-face N6 course*) OR SU (face-to-face N6 course*) | Search modes - Boolean/Phrase | 18 |
| S24 | TI (face-to-face N6 instruct*) OR SU (face-to-face N6 instruct*) | Search modes - Boolean/Phrase | 16 |
| S23 | TI (face-to-face N6 teach*) OR SU (face-to-face N6 teach*) | Search modes - Boolean/Phrase | 18 |
| S22 | TI(face-to-face N6 learn*) OR SU(face-to-face N6 learn*) | Search modes - Boolean/Phrase | 31 |
| S21 | TI(face-to-face N6 educat*) OR SU(face-to-face N6 educat*) | Search modes - Boolean/Phrase | 48 |
| S20 | TI(face-to-face N6 train*) OR SU(face-to-face N6 train*) | Search modes - Boolean/Phrase | 23 |
| S19 | TI(inperson or in-person or "in person") OR SU(inperson or in-person or "in person") | Search modes - Boolean/Phrase | 7,646 |
| S18 | (MH "Education+") | Search modes - Boolean/Phrase | 814,531 |
| S17 | S1 OR S2 OR S3 OR S4 OR S5 OR S6 OR S7 OR S8 OR S9 OR S10 OR S11 OR S12 OR S13 OR S14 OR S15 OR S16 | Search modes - Boolean/Phrase | 52,769 |
| S16 | TI (ehr or emr or epr or ehmr) OR SU (ehr or emr or epr or ehmr) | Search modes - Boolean/Phrase | 2,772 |
| S15 | TI record* system* OR SU record* system* | Search modes - Boolean/Phrase | 8,510 |
| S14 | TI clinic* information system* OR SU clinic* information system* | Search modes - Boolean/Phrase | 6,265 |
| S13 | TI computer* patient record* OR SU computer* patient record* | Search modes - Boolean/Phrase | 86 |
| S12 | TI computer* hospital record* OR SU computer* hospital record* | Search modes - Boolean/Phrase | 0 |
| S11 | TI computer* medical record* OR SU computer* medical record* | Search modes - Boolean/Phrase | 54 |
| S10 | TI computer* health record* OR SU computer* health record* | Search modes - Boolean/Phrase | 3 |
| S9 | TI hospital* information system* OR SU hospital* information system* | Search modes - Boolean/Phrase | 3,001 |
| S8 | TI health information system* OR SU health information system* | Search modes - Boolean/Phrase | 3,085 |
| S7 | TI electronic hospital medical record* OR SU electronic hospital medical record* | Search modes - Boolean/Phrase | 0 |
| S6 | TI electronic health medical record* OR SU electronic health medical record* | Search modes - Boolean/Phrase | 0 |
| S5 | TI electronic patient record* OR SU electronic patient record* | Search modes - Boolean/Phrase | 286 |
| S4 | TI electronic hospital record* OR SU electronic hospital record* | Search modes - Boolean/Phrase | 2 |
| S3 | TI electronic medical record* OR SU electronic medical record* | Search modes - Boolean/Phrase | 1,658 |
| S2 | TI electronic health record* OR SU electronic health record* | Search modes - Boolean/Phrase | 23,063 |
| S1 | (MH "Health Information Systems+") | Search modes - Boolean/Phrase | 50,507 |

| Friday, November 22, 2019 1:24:18 PM  Interface - EBSCOhost Research Databases Search Screen - Advanced Search Database - ERIC |
| --- |

| **#** | **Query** | **Results** |
| --- | --- | --- |
| S29 | S16 AND S28 | 154 |
| S28 | S17 OR S18 OR S19 OR S20 OR S21 OR S22 OR S23 OR S24 OR S25 OR S26 OR S27 | 153,014 |
| S27 | launch* | 5,279 |
| S26 | rollout | 118 |
| S25 | uptake | 1,163 |
| S24 | ehmr N4 adopt* | 0 |
| S23 | epr N4 adopt* | 0 |
| S22 | emr N4 adopt* | 10 |
| S21 | ehr N4 adopt* | 23 |
| S20 | system* N4 adopt* | 1,442 |
| S19 | tech* N4 adopt* | 2,337 |
| S18 | inaugurat* | 466 |
| S17 | implement* | 144,639 |
| S16 | S1 OR S2 OR S3 OR S4 OR S5 OR S6 OR S7 OR S8 OR S9 OR S10 OR S11 OR S12 OR S13 OR S14 OR S15 | 923 |
| S15 | (ehr or emr or epr or ehmr) | 719 |
| S14 | (record* system*) AND (hospital* or health* or medic* or nurs* or patient* or clinic*) | 116 |
| S13 | clinic* information system* | 11 |
| S12 | computer* patient record* | 3 |
| S11 | computer* hospital record* | 1 |
| S10 | computer* medical record* | 5 |
| S9 | computer* health record* | 2 |
| S8 | hospital* information system* | 9 |
| S7 | health information system* | 32 |
| S6 | electronic hospital medical record* | 0 |
| S5 | electronic health medical record* | 0 |
| S4 | electronic patient record* | 3 |
| S3 | electronic hospital record* | 0 |
| S2 | electronic medical record* | 74 |
| S1 | electronic health record* | 107 |
